# Breast reconstruction and breast cancer surgery in Brazil’s public health system: a nationwide interrupted time-series analysis, 2008–2024

**DOI:** 10.1186/s12905-026-04666-x

**Published:** 2026-08-05

**Authors:** Matheus Piccin Padilla, Marina Martins Siqueira, Felipe Mendes Delpino, Julia Baroni Jardim de Freitas, Sergio Podgaec, Felipe Eduardo Martins de Andrade

**Affiliations:** 1 Faculdade Israelita de Ciências da Saúde Albert Einstein, São Paulo, SP Brazil; 2 Hospital Israelita Albert Einstein, São Paulo, SP Brazil

**Keywords:** Breast Neoplasms, Mastectomy, Mammaplasty, Health Policy, Healthcare Disparities, Time-Series Studies, Brazil

## Abstract

**Background:**

Breast reconstruction is a key component of comprehensive breast cancer care, yet access remains uneven in many middle-income countries. In Brazil, federal policies have sought to expand reconstruction within the public health system (SUS), but long-term national trends remain insufficiently characterized. This study aimed to evaluate temporal trends in breast cancer-related surgical procedures and breast reconstruction hospital admissions in Brazil and to examine temporal changes associated with major federal policy measures.

**Methods:**

We conducted a nationwide ecological interrupted time-series study using administrative data from the Brazilian Hospital Information System (SIH/DATASUS) between 2008 and 2024. Annual aggregated hospital admissions for breast cancer-related surgical procedures and breast reconstruction funded by SUS were used as the unit of analysis. The primary outcome was the annual ratio of breast reconstruction hospital admissions to breast cancer-related surgical hospital admissions. Interrupted time-series analyses were performed using segmented negative-binomial regression models.

**Results:**

Between 2008 and 2024, 312,375 hospital admissions for breast cancer-related surgical procedures and 30,049 breast reconstruction hospital admissions were recorded in the Brazilian public health system. The annual breast reconstruction admission ratio increased from 6.67% in 2008 to 19.23% in 2024, corresponding to a significant annual increase of 6.0% (*p* < 0.001). Temporal changes were observed following both Law No. 12,802/2013 and Ordinance No. 127/2023. Despite this increase, breast reconstruction hospital admissions remained substantially lower than breast cancer-related surgical admissions throughout the study period, and marked regional disparities persisted.

**Conclusions:**

Breast reconstruction hospital admissions increased substantially in Brazil’s public health system between 2008 and 2024. Temporal changes coincided with major federal policy measures; however, the persistent gap between breast reconstruction and breast cancer-related surgical hospital admissions suggests continuing challenges in access to reconstructive care. Ongoing monitoring and health policies remain important to improve equitable access to breast reconstruction within SUS.

**Supplementary Information:**

The online version contains supplementary material available at https://doi.org/10.1186/s12905-026-04666-x.

## Background

Breast cancer is the most prevalent malignancy among women in Brazil and a leading cause of cancer-related mortality, with an estimated 73,610 new cases expected annually for the 2023–2025 triennium [1]. Mastectomy, a common and often necessary surgical intervention, can have profound psychosocial consequences, affecting body image, self-esteem, and quality of life [2, 3]. Consequently, breast reconstruction is recognized not merely as a cosmetic procedure, but as an integral component of comprehensive cancer care and a key step in patients’ physical and emotional rehabilitation [4].

In Brazil, the right to breast reconstruction has been legally guaranteed within the Unified Health System (SUS) since 1999, with the enactment of Law No. 9,797 [5]. This right was further reinforced by Law No. 12,802 in 2013, which established that reconstruction should be performed immediately following mastectomy whenever technically feasible [6]. More recently, in response to persistently low reconstruction ratios, the Ministry of Health issued Ordinance No. 127 in February 2023, expanding access and increasing funding for these procedures within the SUS [7].

Despite this legal framework, access to breast reconstruction remains limited and uneven across the country. Previous studies, such as Almeida et al. (2021), have provided important insights by analyzing data from 2015 to 2020 [8]. However, these analyses are constrained by shorter timeframes and do not capture long-term trends or the effects of recent policy changes. In addition, the COVID-19 pandemic significantly disrupted elective procedures, including mastectomies, breast-conserving surgeries and reconstructions, creating a backlog and reshaping the healthcare landscape in ways that are not yet fully understood [9].

To address these gaps, this study presents a comprehensive and up-to-date analysis of breast cancer-related surgical procedures and breast reconstruction ratios within the Brazilian public health system. Using a 17-year time series (2008–2024), it aims to describe long-term trends in surgical management and reconstruction, as well as to evaluate temporal changes associated with key public policies across Brazil and its regions. By providing this longitudinal perspective, the study seeks to generate robust evidence to inform future health policies and contribute to efforts to ensure equitable access to comprehensive breast cancer care in Brazil.

## Methods

### Study design and setting

This nationwide retrospective ecological time-series study used routinely collected administrative data from the Brazilian Unified Health System (Sistema Único de Saúde—SUS). We analyzed hospitalizations recorded between January 2008 and December 2024, encompassing a 17-year period across Brazil. The unit of analysis was the annual aggregated volume of hospital admissions for breast cancer-related surgical procedures and breast reconstruction within SUS-funded care. Because SIH/DATASUS is an administrative database based on hospital admissions, individual patients could not be identified or linked across multiple admissions.

### Data sources and linkage

Data were obtained from the Hospital Information System (SIH/DATASUS), a nationwide administrative database maintained by the Brazilian Ministry of Health that records all hospital admissions reimbursed by SUS. Hospital admissions were identified using procedure codes from the SUS Unified Procedure Table (SIGTAP) [10] and aggregated by year, geographic region, and procedure type. Population estimates were obtained from the Brazilian Institute of Geography and Statistics (IBGE) [11] and were used to calculate population-adjusted rates. Geographic displacement was assessed using DATASUS variables for the municipality of residence and the municipality of hospitalization.

Data completeness was assessed for all variables included in the analyses. No missing data were identified for age, sex, municipality of residence, municipality of hospitalization, ICD-10 diagnosis codes, procedure costs, or hospital identifiers in the SIH/DATASUS database. Hospital accreditation information was obtained through deterministic linkage with the National Registry of Health Establishments (CNES) using the unique establishment identifier (CNES code). This linkage was successful for all hospital admissions, and no records were excluded because of missing information.

### Study sample

We included all hospital admission records for breast cancer-related surgical procedures and breast reconstruction funded by SUS between January 2008 and December 2024. No restrictions were applied regarding age or sex, and no additional exclusion criteria were defined.

## Definition of surgical procedures

The following procedure codes related to the surgical management of breast cancer and breast reconstruction were included.

Codes for surgical management of breast cancer:

- 0410010057 – Radical mastectomy with lymphadenectomy.
- 0410010065 – Simple mastectomy.
- 0416120024 – Radical mastectomy with axillary lymphadenectomy in oncology.
- 0416120032 – Simple mastectomy in oncology.
- 0410010111 – Segmentectomy/quadrantectomy.
- 0410010120 – Segmentectomy/quadrantectomy with lymph node dissection.
- 0416120059 – Segmentectomy/quadrantectomy/setorectomy in oncology.

Codes for breast reconstruction procedures:

- 0410010090 – Post-mastectomy breast reconstruction with implant.
- 0410010073 – Non-aesthetic female breast reconstruction.
- 0410010219 – Breast reconstruction after total mastectomy.

Following Ordinance No. 127/2023, the SIGTAP procedure code 0410010219 (“Post-total mastectomy breast reconstruction”) became available for reimbursement within SIH/SUS and was incorporated into the identification of breast reconstruction hospital admissions from 2023 onward [7].

For the purposes of this study, the term “breast cancer-related surgical procedures” refers collectively to simple and radical mastectomy, segmentectomy, quadrantectomy, and their corresponding oncologic procedure codes listed above. This definition was applied consistently throughout the manuscript unless otherwise specified.

To ensure oncological specificity, all records were filtered by International Classification of Diseases, 10th Revision (ICD-10) diagnostic codes corresponding to malignant neoplasm of the breast (C50) and carcinoma in situ of the breast (D05) [12].

### Variables

#### Outcomes

- The primary outcome was the annual ratio of breast reconstruction admissions to breast cancer-related surgical admissions, expressed as a percentage.
- Breast cancer-related surgical procedures were selected as the denominator because reconstructive procedures may follow different types of breast cancer surgery, including mastectomy and breast-conserving procedures. In addition, because SIH/DATASUS records hospital admissions rather than longitudinal patient-level data, reconstruction admissions cannot be linked to the corresponding oncologic procedure. Therefore, this denominator was chosen to provide a comprehensive population-level indicator of reconstructive activity within the Brazilian public health system rather than an estimate of post-mastectomy reconstruction at the individual level. This methodological approach was defined a priori in consultation with breast surgery specialists.
- Population-adjusted hospitalization rates for breast cancer surgical management and breast reconstruction were expressed per 100,000 female inhabitants by region.

#### Geographic and hospital characteristics

- Regional disparities in access to care were assessed by the travel distance (in kilometers), estimated as the straight-line distance between the centroid of the patient’s municipality of residence recorded for each hospital admission and the geographic location of the hospital where the corresponding procedure was performed. Distances were calculated for each hospital admission and summarized as regional mean and median values.
- Hospital characteristics were analyzed for the year 2024 and categorized as specialized oncology centers (CACON/UNACON) or non-specialized hospitals according to the official Ministry of Health accreditation registry, based on the oncology accreditation status assigned to each hospital under Ordinance No. 140/2014. Accreditation was assessed at the hospital level. Hospitals without CACON or UNACON accreditation were classified as non-specialized hospitals. Because SIH/DATASUS includes all hospital admissions reimbursed by SUS, the analysis encompasses all public hospitals performing breast cancer-related surgical procedures and breast reconstruction within the public health system, regardless of accreditation status. The distribution of specialized centers was further described according to their location in capital cities versus non-capital municipalities [13].

####  Economic variables

- Economic variables included the mean cost per hospitalization by procedure type and cumulative costs over the study period. Demographic variables comprised patient sex and age, summarized as proportions and mean values, respectively.

#### Policy evaluation

- The legislative periods were defined based on the enactment of Law No. 12,802/2013 and Ordinance No. 127/2023 [6, 7].

### Data analysis

We assessed trends in the ratio of breast reconstruction hospital admissions to breast cancer-related surgical hospital admissions with a segmented (interrupted time series) regression on annual counts. We used annual rather than monthly data because the source records procedures by year of competence, and monthly counts for these procedure codes were sparse and affected by reporting lags that distort short windows; yearly aggregation also removes seasonality. The resulting series is short, which is why we relied on the autocorrelation diagnostics and sensitivity analyses described below rather than on standard errors alone.

We modelled the annual count of breast reconstruction admissions with a log link and the natural logarithm of the annual number of breast cancer-related surgical admissions as an offset, so the outcome was the reconstruction ratio among those procedures:

log E(Yt) = β₀ + β₁ · timet + β₂ · levelt + β₃ · trendt + log(Nt).

Here time was coded 0 in the first year of the series (the reference year, 2008) and increased by one each year; level was 0 before 2013 and 1 from 2013 onward; and trend counted the years since the intervention (0 before; 1, 2, 3 … from 2013). Under this coding, exp(β₁) is the pre-intervention annual rate ratio, exp(β₂) the immediate change in level at 2013, and exp(β₃) the change in the annual trend thereafter, with the post-intervention trend equal to exp(β₁ + β₃). Estimates are reported as incidence rate ratios (IRR) with 95% confidence intervals. Analyses were performed in R (version X.X.X; R Foundation for Statistical Computing, Vienna, Austria).

We first fitted a Poisson model and assessed overdispersion from the Pearson dispersion statistic and by comparison with a negative-binomial model. Overdispersion was substantial (Pearson dispersion ≈ 42) and the negative-binomial model was strongly preferred (AIC 243 vs. 738), so the negative-binomial specification was used for the main analysis, with quasi-Poisson estimation as a robustness check. Model fit was summarised by the AIC and the residual deviance.

We examined residual autocorrelation from the autocorrelation (ACF) and partial autocorrelation (PACF) functions of the deviance residuals and the Durbin–Watson statistic. Because robust standard errors are themselves unreliable in a short series, inference was based on the negative-binomial model rather than on robust errors alone; heteroskedasticity- and autocorrelation-consistent (Newey–West) standard errors were also computed as a check. Influential years were screened with Cook’s distance (4/n threshold).

We assessed robustness by re-fitting the model after excluding the pandemic years (2020–2022) and after shifting the intervention point by ± 1 year to test its assumed timing.

### Ethical considerations

This study used publicly available anonymized secondary data. In accordance with Resolution No. 510/2016 of the Brazilian National Health Council, ethics committee approval was not required [14].

## Results

### Record selection and study sample

Over the 17-year study period (2008–2024), a total of 312,375 hospital admissions for breast cancer-related surgical procedures and 30,049 for breast reconstruction were identified and included in the analysis. Of the 312,375 hospital admissions for breast cancer-related surgical procedures, 289,813 (92.8%) were associated with invasive breast cancer (ICD-10 C50) and 22,562 (7.2%) with carcinoma in situ (ICD-10 D05). Breast reconstruction admissions were almost exclusively associated with invasive disease, with 29,947 (99.7%) coded as C50 and only 101 (0.3%) coded as D05 (Supplementary Table S3). Among admissions for breast cancer surgical management, 98.4% were female, with an average age of 54.4 years. For breast reconstruction, 99.97% of admissions were female, with a mean age of 49.7 years.

### Descriptive data and overall rates

**Fig. 1:**
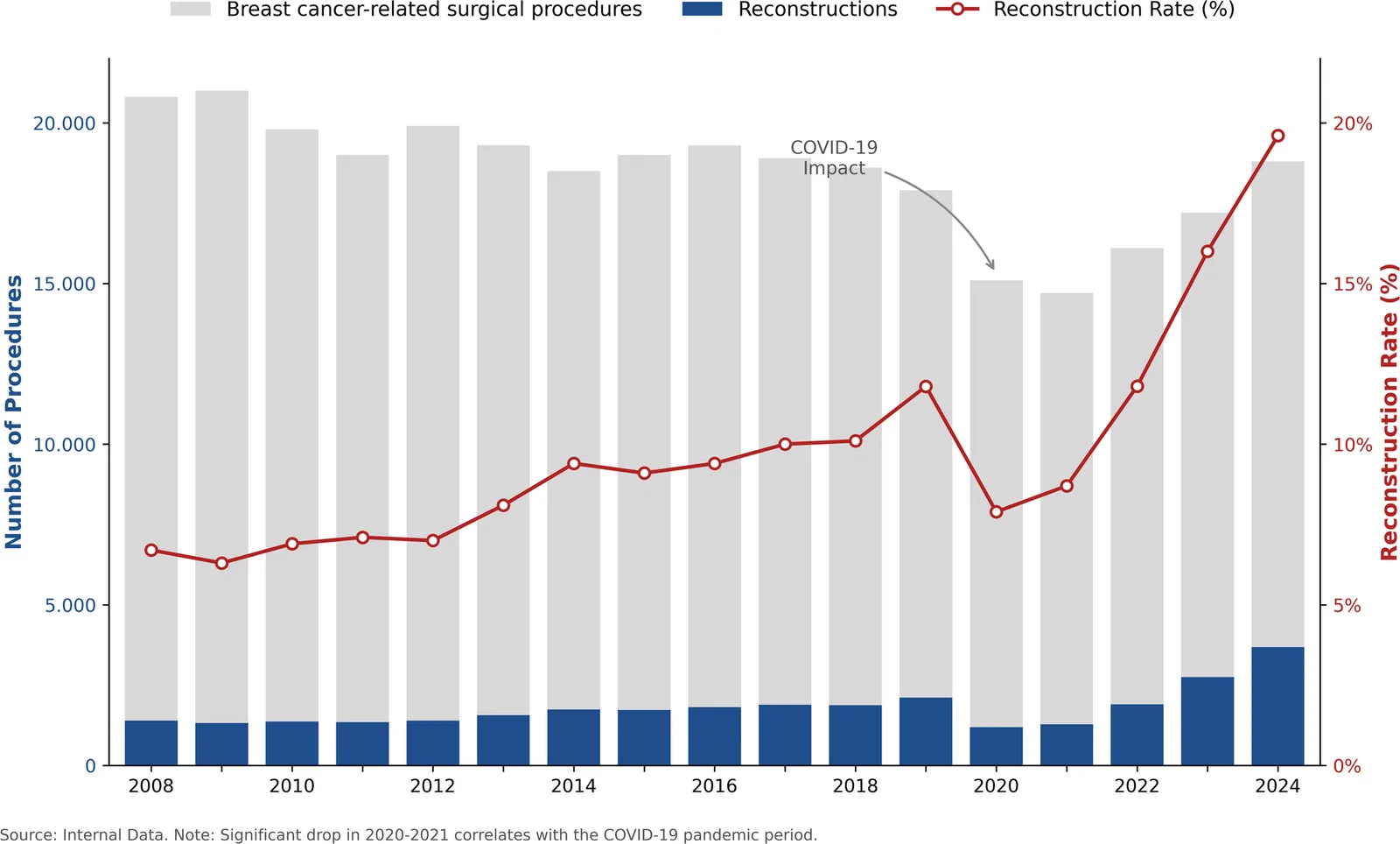
Evolution of breast reconstruction ratio (2008–2024)

The overall breast reconstruction ratio was 9.62% across the study period. On average, there were 18,375 procedures for breast cancer surgical management and 1,768 reconstruction procedures annually. Detailed yearly data are shown in Fig. 1. The reconstruction ratio increased significantly over time, with an average annual growth of 6.00% (*p* < 0.001), rising from 6.6% in 2008 to 19.2% in 2024.

The mean approved reimbursement per hospital admission increased over the study period. For breast cancer-related surgical procedures, the mean reimbursement increased from R$1,569.39 (US$291.17) in 2008 to R$2,564.73 (US$475.83) in 2024, with an overall mean of R$2,606.14 (US$483.51). For breast reconstruction, the mean reimbursement increased from R$2,043.56 (US$379.14) in 2008 to R$3,403.51 (US$631.45) in 2024, with an overall mean of R$1,680.75 (US$311.83). Detailed annual reimbursement values in inflation-adjusted 2024 Brazilian reais and corresponding constant 2024 US dollars are presented in Supplementary Table S2.

Over the 17-year period, total expenditures for hospitalizations related to breast cancer surgical management reached R$ 814,094,168.78 ( U$ 151,037,879.18), while breast reconstruction procedures accounted for R$ 50,503,277.46 (U$ 9,369,810.29) within the public health system.

Monetary values were based on the approved reimbursement amounts recorded in SIH/DATASUS for each hospital admission, including both hospital and professional service components. Annual mean reimbursement values were calculated by dividing the total approved reimbursement by the corresponding number of hospital admissions for each procedure category. Nominal reimbursement values were adjusted for inflation to constant 2024 Brazilian reais using the accumulated National Consumer Price Index (IPCA). For each year, the cumulative inflation factor corresponding to the period up to 2024 was applied to ensure comparability in real terms. Inflation-adjusted values were subsequently converted to constant 2024 US dollars using the average commercial exchange rate for 2024 (US$1 = R$5.39). No hospital admissions included in the analyses had missing reimbursement values [15, 16].

### Regional aspects and geographic disparities

The Southeast region accounted for the highest absolute numbers of hospitalizations, representing 49% of hospitalizations for breast cancer surgical procedures and 63% of breast reconstruction over the study period. In contrast, the North contributed the smallest share, with 5% and 2% of hospitalizations for breast cancer surgery and reconstruction, respectively.

After adjusting for population size, hospitalization rates were expressed per 100,000 women. In 2008, the North had the lowest rate of breast cancer surgical procedures (4.82), whereas the South (19.46) and Southeast (18.01) had the highest rates. By 2024, the rate in the North had increased to 9.55, while rates in the South (13.58) and Southeast (17.20) showed a declining trend.

For breast reconstruction procedures, all regions experienced increases in hospitalization rates over the 17-year period. The North region, which had the lowest rate in 2008 (0.03), increased to 0.88 by 2024. The Southeast (from 1.57 to 3.73) and South (from 1.73 to 3.33) also showed substantial increases and remained among the highest rates nationwide (Table 1).

**Table 1:** Regional hospitalization rates per 100,000 inhabitants (2008 vs. 2024)

Region	Breast cancer surgical procedures (2008)	Breast cancer surgical procedures (2024)	Reconstruction (2008)	Reconstruction (2024)
North	4.82	9.55	0.03	0.88
Northeast	10.37	7.79	0.23	1.22
Southeast	18.01	17.20	1.57	3.73
South	19.46	13.58	1.73	3.33
Central-West	10.63	10.41	0.50	1.08
Brazil	13.89	12.39	0.93	2.38

To support regional analyses, hospital characteristics were assessed for the most recent year (2024). Among breast cancer surgical procedures, 16,395 of 18,706 (87.6%) were performed in specialized centers (CACON/UNACON). These centers also accounted for 3,437 of 3,597 breast reconstruction procedures (95.5%) in the same year.

**Fig. 2:**
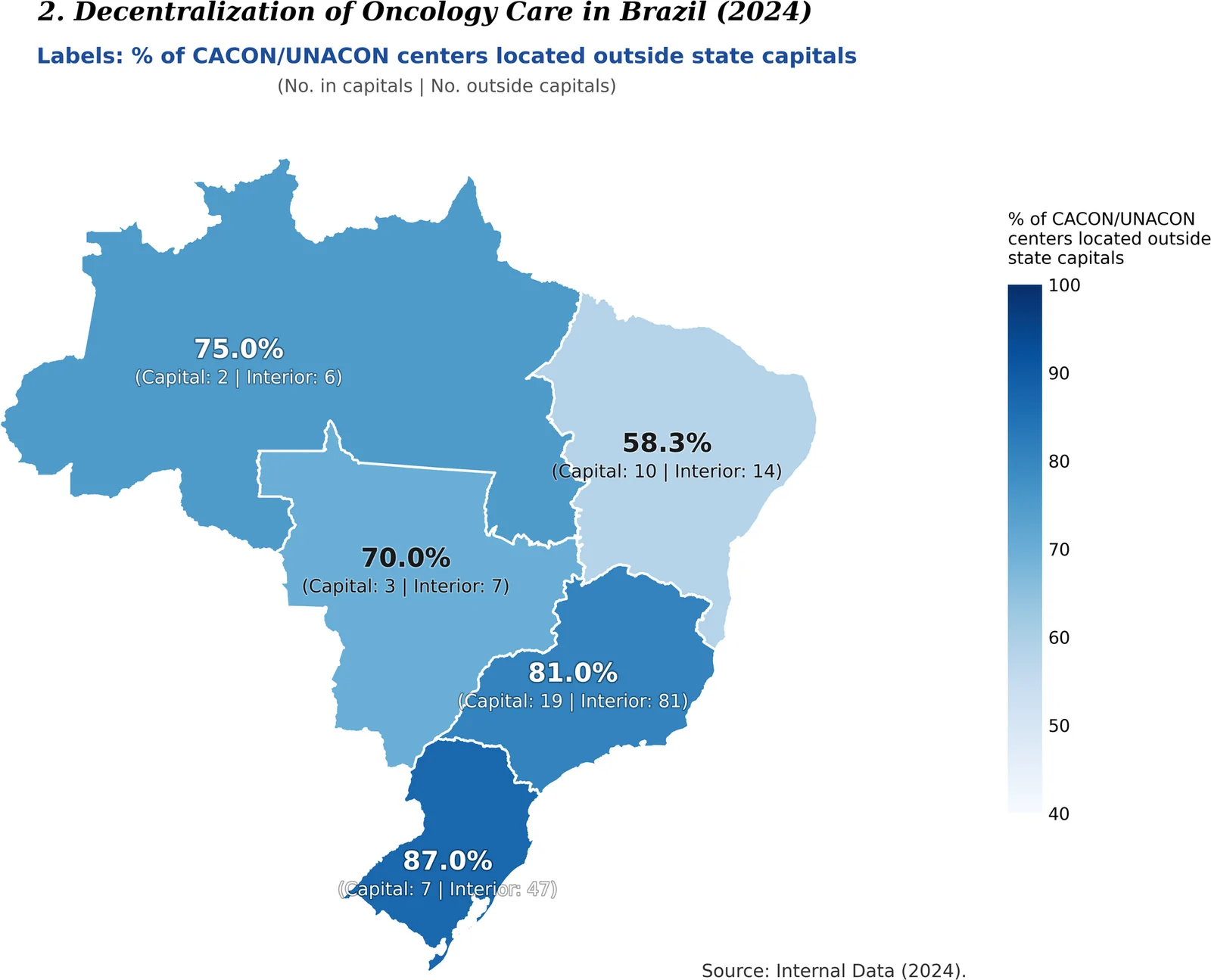
Decentralization of Oncology Care in Brazil (2024)

The distribution of these specialized centers varied across Brazil’s macro-regions. In the South and Southeast, most centers were located outside capital cities, with only 13% and 19% situated in capitals, respectively. In contrast, other regions showed a higher concentration in capital cities, particularly the Northeast, where 41% of centers were located in capitals, the highest proportion nationwide (Fig. 2).

Geographic access to care was assessed by estimating the distance between the patient’s municipality of residence and the hospital where the procedure was performed. In 2024, mean travel distances for both breast cancer surgical procedures and breast reconstruction were highest in the North and Northeast, exceeding 150 km in both regions, whereas shorter distances were observed in the South and Southeast regions, with mean values below 80 km.

Median distances showed a similar pattern. In the North region, the median exceeded 100 km, and in the Northeast it approached 90 km, while in the South and Southeast median distances remained below 45 km. These regional disparities persisted throughout the study period, with limited variation over time (Table 2).

**Table 2:** Mean and median distance to treatment centers by region (km)

Region	Mean Distance (km)	Median Distance (km)
North	172.98	115.08
Northeast	150.33	90.50
Central-West	134.48	113.61
South	73.10	42.66
Southeast	53.83	31.22
Brazil	91.57	40.62

### Temporal trends following major public policies

Analyses were stratified by legislative period, using Law No.12,802/2013 and Ordinance No. 127/2023 as reference points. The mean breast reconstruction ratio increased 6.78% before the 2013 law to 9.61% after its implementation, and to 17.63% following the 2023 ordinance. The ITS analysis, adjusted using robust standard errors, showed that Law No. 12,802/2013 was associated with an immediate relative increase of 20.4% in the reconstruction ratio (*p* < 0.001). The 2023 ordinance was associated with a larger immediate increase of 46.7% (*p* < 0.001), as well as a significant change in trend, corresponding to an additional annual increase by 18.5% per year (*p* < 0.001). By 2024, the observed breast reconstruction ratio had reached 19.23% (Table 3).

**Table 3:** Breast reconstruction ratios and estimated Temporal trends of legislative interventions (2008–2024)

Legislative Period	Years	Mean Observed Rate (%)	Immediate Relative Impact (95% CI)	Annual Trend Change (95% CI)
Pre-Law 12,802	2008–2012	6.78	Reference	Reference
Post-Law 12,802	2013–2022	9.61	+ 20.4% (13.2–28.1)*	-0.2% (-2.0–1.5)
Post-Ordinance 127	2023–2024	17.63	+ 46.7% (39.5–54.4)*	+ 18.5% (12.7–24.6)*

*CI* confidence interval

¹ Immediate relative increase compared to the previous trend (level change)

² Change in annual growth rate (slope change)

*p < 0.001

### Sensitivity analyses

To evaluate whether the COVID-19 pandemic influenced the estimated effects of the 2023 policy intervention, sensitivity analyses were performed by excluding the pandemic years (2020–2022) and by shifting the intervention point by ± 1 year (Table 4). The main findings were consistent across all sensitivity analyses (Supplementary Table S1). The immediate level change at the intervention remained non-significant across all model specifications (IRR 0.99–1.05; all *p* > 0.60). The estimated post-intervention trend remained positive in every analysis (IRR 1.02–1.04). In the primary analysis, the trend change did not reach statistical significance (IRR 1.03, 95% CI 0.94–1.12; *p* = 0.52), whereas exclusion of the COVID-19 pandemic years resulted in a statistically significant positive trend change (IRR 1.04, 95% CI 1.01–1.08; *p* = 0.008). This finding suggests that the increased variability during the pandemic period may have reduced statistical precision without altering the overall direction of the temporal trend. Likewise, shifting the intervention point by one year in either direction produced similar estimates for both the immediate level change and the post-intervention trend, supporting the robustness of the model assumptions (Supplementary Table S1).

**Table 4:** Sensitivity analyses of the interrupted time-series model evaluating the 2023 policy intervention

Analysis	Years (*n*)	Level change, IRR (95% CI)	Trend change, IRR (95% CI)
Main analysis (full series)	17	1.05 (0.80–1.36), *p* = 0.74	1.03 (0.94–1.12), *p* = 0.52
Excluding pandemic years (2020–2022)	14	1.03 (0.93–1.13), *p* = 0.61	1.04 (1.01–1.08), *p* = 0.008
Intervention lag, − 1 year	17	0.99 (0.75–1.30), *p* = 0.94	1.03 (0.91–1.16), *p* = 0.69
Intervention lag, + 1 year	17	1.02 (0.79–1.32), *p* = 0.88	1.02 (0.95–1.09), *p* = 0.64

*IRR* incidence rate ratio, *CI* confidence interval

Estimates were obtained from segmented negative-binomial regression models using the natural logarithm of the annual number of breast cancer-related surgical admissions as the offset term. Level change represents the immediate change in the breast reconstruction admission ratio following the 2023 policy intervention, whereas trend change represents the change in the annual post-intervention trend. The negative-binomial model was selected because the Poisson model showed substantial overdispersion (Pearson dispersion ≈ 42; AIC 738 vs. 243). Quasi-Poisson estimation yielded consistent conclusions.

An additional sensitivity analysis restricted to hospital admissions coded as invasive breast cancer (ICD-10 C50) yielded results that were virtually identical to those of the primary analysis, with no material changes in the estimated level or trend effects (Supplementary Table S5). These findings indicate that inclusion of carcinoma in situ (ICD-10 D05) did not materially influence the study results.

### Procedure coding after Ordinance No. 127/2023

**Fig. 3:**
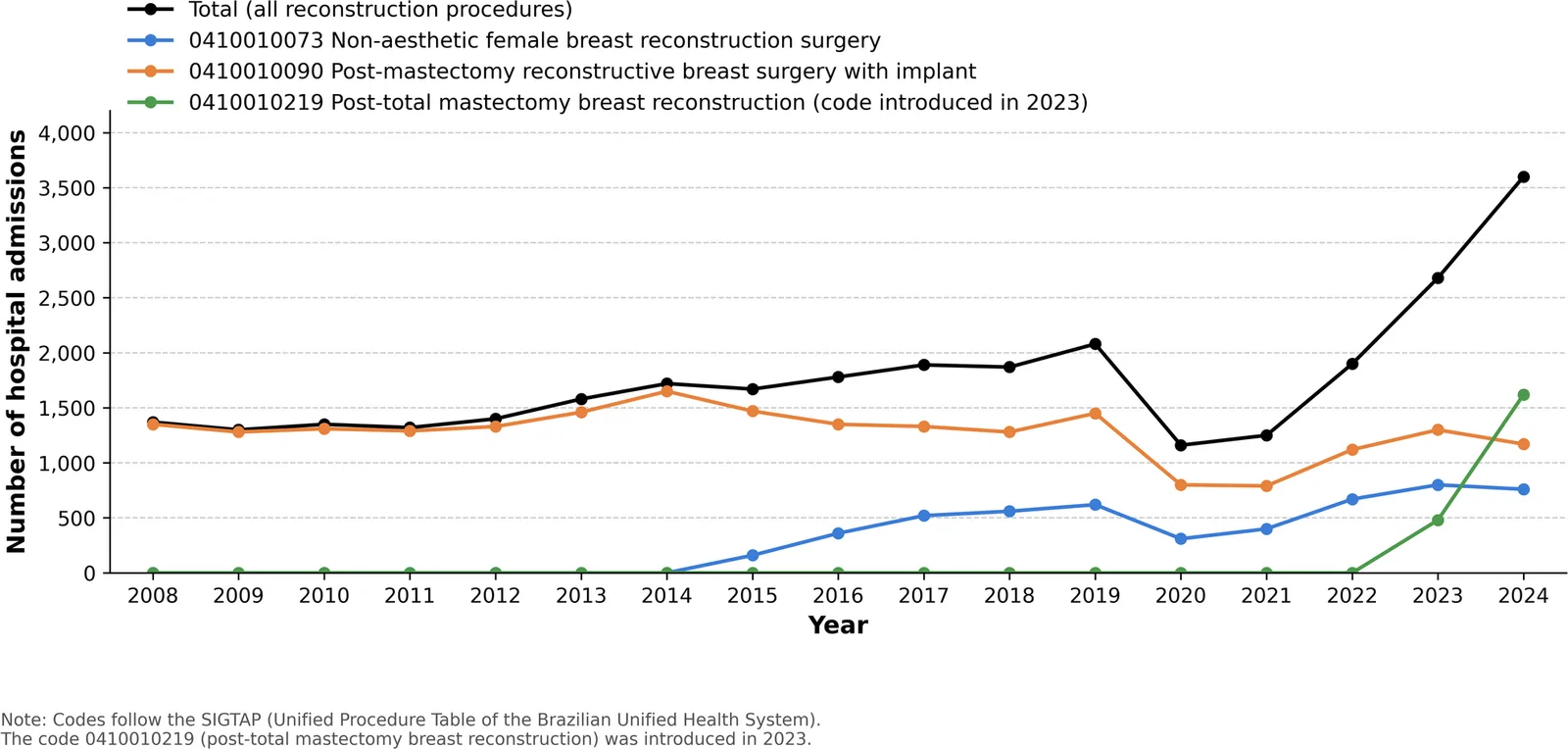
Annual volume of breast reconstruction hospital admissions according to SIGTAP procedure code, Brazil, 2008–2024

Following the implementation of Ordinance No. 127/2023, a new SIGTAP procedure code (0410010219, “Post-total mastectomy breast reconstruction”) was introduced into SIH/SUS. Figure 3 presents the annual distribution of breast reconstruction hospital admissions according to individual procedure codes. Before 2023, breast reconstruction hospital admissions were recorded using the previously available reconstruction procedure codes. After the introduction of the new code, hospital admissions were distributed across all three reconstruction procedure codes, with the new procedure code accounting for an increasing proportion of admissions while the previously existing codes remained in use throughout 2023 and 2024. The corresponding annual numbers of hospital admissions are presented in Supplementary Table S1.

## Discussion

To our knowledge, this study represents the largest nationwide analysis of hospital admissions for breast cancer-related surgical procedures and breast reconstruction within Brazil’s public health system, covering a 17-year period that includes key events such as legislative changes and the COVID-19 pandemic. Overall, the findings indicate a sustained increase in the breast reconstruction ratio, accompanied by a parallel rise in the volume of breast cancer-related surgical admissions. Notably, a more pronounced change in trend was observed following the implementation of federal policy incentives.

The 2013 law and the 2023 ordinance approach breast reconstruction through different regulatory mechanisms [6, 7]. The 2013 law established the right to immediate reconstruction when technically feasible but did not define specific mechanisms for implementation, financing, service accreditation, or monitoring. In contrast, the 2023 ordinance operates at administrative level, introducing targeted funding, improving traceability, and reinforcing the role of specialized oncology centers (CACON/UNACON).

The 2023 ordinance also incorporated the procedure “breast reconstruction after total mastectomy” into the SUS reimbursement system (SIGTAP), assigning a value of U$ 1,047.89 per procedure (U$ 855.82 for hospital services and U$ 192.06 for professional services). By comparison, the ‘post-mastectomy breast reconstruction with implant’ procedure, available since 2008, currently has a reimbursement value of U$ 58.61.These discrepancies highlight a broader structural issue: reimbursement values within the SUS are often outdated and may not reflect the actual costs of care. Financial incentives embedded in reimbursement mechanisms may also influence the provision of reconstructive procedures. When procedure codes and funding streams are clearly defined, healthcare providers may be more likely to incorporate reconstruction into routine oncologic care.

The observed increase in breast reconstruction hospital admissions after 2023 should also be interpreted in the context of changes in administrative procedure coding. Ordinance No. 127/2023 introduced a new SIGTAP procedure code specifically for post-total mastectomy breast reconstruction, which became operational within SIH/SUS in 2023. Analysis of procedure-specific hospital admissions showed that this new code was progressively adopted, while the previously available reconstruction procedure codes remained in use during 2023 and 2024. Therefore, although the introduction of the new code may have improved the recording and classification of reconstructive procedures in administrative data, the available data do not allow distinguishing the relative contributions of changes in coding practices and a true expansion in reconstructive activity to the increase observed after 2023.

Although no adjustments were made to reimbursement values for reconstruction after total mastectomy in 2024, Ordinance No. 1693 (October 5, 2024) expanded eligibility criteria, allowing these procedures to be performed in patients of both sexes [17].

Taken together, these findings suggest that the impact of the 2013 law appears smaller than that observed after the 2023 ordinance, with immediate relative increase of 20.4% and 46.7%, respectively. This more modest effect may reflect a common challenge in health policy implementation: the formal recognition of rights does not necessarily translate into effective service delivery without supporting regulatory frameworks, financing mechanisms, and operational guidelines.

The period between 2020 and 2022 coincided with a global reduction in elective surgical procedures due to the COVID-19 pandemic, likely generating pent-up demand in the subsequent years. In addition, the post-ordinance period includes only two years (2023–2024), limiting the ability to assess long-term trend changes and the sustainability of the observed increase in reconstruction ratio. Despite these limitations, the post-ordinance period shows not only an immediate increase but also a significant change in trend, with an additional annual growth of 18.5%. This pattern suggests a possible shift in the temporal trajectory of reconstruction ratio within the public health system.

Moreover, marked regional differences were observed in the travel distances associated with hospital admissions for breast cancer-related surgical procedures and breast reconstruction. In 2024, hospital admissions involving residents of the North and Northeast were associated with substantially longer travel distances, with mean values exceeding 150 km and median distances close to or above 90 km. In contrast, hospital admissions involving residents of the South and Southeast were associated with shorter travel distances, with means below 80 km and medians under 45 km. These geographic patterns were descriptively concordant with the regional differences observed in breast reconstruction ratios; however, formal statistical analyses of this relationship were not performed.

These patterns were accompanied by consistent differences in reconstruction ratios. Regions characterized by longer distances exhibited persistently lower reconstruction ratios, whereas those with shorter distances maintained higher rates throughout the study period. Although causality cannot be established, this parallel suggests that geographic distance may act as a relevant barrier to access within the SUS.

Longer travel distances may restrict access to specialized surgical teams, increase indirect costs for patients and their families, and complicate postoperative follow-up. These factors have been previously described as barriers to breast reconstruction in different healthcare settings [8, 18, 19]. While oncologic surgeries such as mastectomy and breast-conserving procedures are often unavoidable, breast reconstruction may be more sensitive to these constraints, particularly when referral to specialized centers is required.

In the Brazilian context, the concentration of CACON and UNACON facilities in capital cities further reinforces this pattern, disproportionately affecting patients from rural and remote areas. Together, these findings suggest that regional disparities in reconstruction ratios are not solely driven by clinical or legal factors, but are closely linked to the spatial organization of specialized oncology services. Because information on hospital accreditation was evaluated only for 2024, these results should be interpreted as a contemporary snapshot of the organization of oncology services rather than as an explanation for the temporal trends observed over the 17-year study period.

Beyond the geographic distribution of services, the availability of trained reconstructive surgeons and multidisciplinary oncology teams can also influence reconstruction ratios. The concentration of specialized surgical expertise in large urban centers is a well-described structural feature of the Brazilian health system and may further contribute to regional inequalities in access to reconstructive procedures [20]. 

From a policy perspective, these findings underscore the importance of territorial planning in the organization of oncologic care within the SUS. Strategies such as expanding reconstructive capacity in regional centers, strengthening referral networks, and developing specialized surgical outreach programs may help reduce geographic barriers and improve equity in access.

In this context, nationwide administrative databases, as illustrated in this analysis, represent a valuable resource for health system evaluation. Routine health information systems enable the monitoring of public policy effects on surgical care delivery and support periodic assessments of access and regional equity, particularly in large and heterogeneous countries such as Brazil.

These results should be interpreted in light of several limitations. The breast reconstruction ratios observed in the present study are lower than those described in previous national studies, such as Almeida et al. (2021), likely because of methodological differences. Unlike previous studies, which primarily evaluated immediate breast reconstruction performed at the time of mastectomy, our analysis included all breast reconstruction admissions recorded in SIH/DATASUS, irrespective of whether they represented immediate or delayed reconstruction, and considered all breast cancer-related surgical admissions, including mastectomy, segmentectomy, and quadrantectomy, as potential sources of reconstructive demand. This methodological approach, based on a broader denominator, provides a more comprehensive population-level assessment of reconstructive activity within the Brazilian public health system. Although mastectomy is the principal and most predictable indication for breast reconstruction, breast-conserving procedures may also generate reconstructive demand in selected patients. Consequently, using all breast cancer-related surgical admissions as the denominator is likely to underestimate breast reconstruction ratios compared with analyses restricted to mastectomy alone, but better reflects the full spectrum of potential reconstructive demand within the public health system [8]. 

Because SIH/DATASUS is an administrative database based on hospital admissions rather than longitudinal patient-level records, it is not possible to distinguish immediate from delayed breast reconstruction or to link each reconstruction admission to its corresponding oncologic procedure. In addition, SIH/DATASUS does not include detailed tumor-level clinical information, such as tumor stage or molecular subtype. Therefore, it was not possible to evaluate how differences in disease characteristics may have influenced the likelihood of breast reconstruction.

Additional limitations arise from the ecological design and the use of secondary administrative data, which are subject to heterogeneous reporting practices and potential coding errors, including misclassification of ICD-10 diagnoses, procedures, and patient characteristics. Accordingly, the findings should be interpreted as population-level associations rather than estimates at the individual patient level, and causal inferences cannot be established.

Within this framework, the results suggest that federal legislation and administrative interventions have contributed to expanding access to breast reconstruction within the SUS. Recent measures, such as Law No. 15.171/2025, may offer further opportunities to improve access and reduce disparities; however, it remains premature to assess their long-term impact, as well as that of the 2023 ordinance [21]. Continued monitoring and future population-based studies will be essential to determine whether these policies lead to sustained improvements in reconstructive care across regions.

## Conclusions

This nationwide study of hospital admissions within the Brazilian public health system (SUS) describes long-term trends in breast cancer-related surgical procedures and breast reconstruction within the SUS and examines their association with federal policy interventions. Breast reconstruction ratios increased over time, particularly following recent administrative measures; however, substantial regional disparities in geographic access and service availability persist. Although the ecological design limits causal inference, the findings provide important population-level insights into access to reconstructive care. They underscore the need to align legal frameworks, financing mechanisms, and the territorial organization of specialized services to ensure that the right to breast reconstruction translates into equitable access nationwide. Future studies incorporating more granular clinical data are warranted to assess the sustainability of recent policy effects and to inform strategies aimed at reducing disparities in breast reconstruction.

## Supplementary Information

Supplementary Material 1.

Supplementary Material 2.

## Data Availability

The datasets analyzed during the current study are publicly available from the Brazilian Ministry of Health through the DATASUS platform (https://datasus.saude.gov.br/). All data used in this study can be accessed through the public DATASUS repository.

## Abbreviations

ACF: Autocorrelation Function
AIC: Akaike Information Criterion
CACON: High-Complexity Oncology Center (Centro de Assistência de Alta Complexidade em Oncologia)
CI: Confidence Interval
CNES: National Registry of Health Establishments (Cadastro Nacional de Estabelecimentos de Saúde)
COVID-19: Coronavirus Disease 2019
DATASUS: Department of Informatics of the Brazilian Unified Health System
IBGE: Brazilian Institute of Geography and Statistics
ICD-10: International Classification of Diseases, 10th Revision
IPCA: National Consumer Price Index (Índice Nacional de Preços ao Consumidor Amplo)
IRR: Incidence Rate Ratio
ITS: Interrupted Time-Series
PACF: Partial Autocorrelation Function
SIGTAP: Unified Health System Procedure Management System (Sistema de Gerenciamento da Tabela de Procedimentos, Medicamentos e OPM do SUS)
SIH: Hospital Information System (Sistema de Informações Hospitalares)
SUS: Brazilian Unified Health System
UNACON: High-Complexity Oncology Care Unit (Unidade de Assistência de Alta Complexidade em Oncologia)
