# Supplementary material for: Breast reconstruction and breast cancer surgery in Brazil’s public health system: a nationwide interrupted time-series analysis, 2008–2024
Source: BMC Womens Health. 2026 Aug 5;26:574. doi: 10.1186/s12905-026-04666-x (PMC13726083; doi:10.1186/s12905-026-04666-x)
Supplement: Supplementary file 1 [file 12905_2026_4666_MOESM1_ESM.docx]

\# ============================================================================

\# Breast reconstruction after mastectomy in Brazil

\# Descriptive trends and interrupted time series (ITS) analysis

\#

\# Reproducible analysis script

\# Author(s): [add author names]

\#

\# ----------------------------------------------------------------------------

\# HOW TO RUN

\#	1. Place the input file in your working directory (or set INPUT_FILE below

\#	to its full path on your machine).

\#	2. Set the working directory to that folder (Session > Set Working Directory,

\#	or use an RStudio project). Do NOT hard-code personal paths here.

\#	3. Source the whole script. All results and figures are written to ./output.

\#

\# INPUT : one row per hospital procedure record. The relevant columns (year,

\#	region, procedure description) are named in the SETTINGS block and

\#	follow SIH-SUS/DATASUS exports; adjust the names to match your file.

\# OUTPUT : ./output/reconstruction_results.xlsx (all tables)

\#	./output/fig_acf_pacf.png	(residual autocorrelation)

\#	./output/fig_its_fit.png	(observed / fitted / counterfactual)

\#	./output/sessionInfo.txt	(R and package versions)

\#

\# METHODS NOTE

\#	The outcome is the annual count of breast reconstruction procedures, modelled

\#	with a log link and an offset of log(total mastectomy-related procedures).

\#	The series is interrupted at INTERVENTION_YEAR with a level term and a trend

\#	(slope-change) term. The Poisson model is over-dispersed, so the NEGATIVE

\#	BINOMIAL model is the main specification; Poisson, quasi-Poisson and

\#	Newey-West (HAC) estimates are reported as robustness checks. In a short

\#	annual series the HAC standard errors are themselves unreliable and are not

\#	used as the basis for inference.

\# ============================================================================

rm(list = ls())

\# ---------------------------------------------------------------------------

\# 0) Packages

\# ---------------------------------------------------------------------------

if (!requireNamespace("pacman", quietly = TRUE)) install.packages("pacman")

pacman::p_load(tidyverse, readxl, writexl, stringi, MASS)

\# Note: MASS masks dplyr::select(); call dplyr::select() explicitly if needed.

\# ---------------------------------------------------------------------------

\# 1) SETTINGS (edit these; no personal paths committed to the public repo)

\# ---------------------------------------------------------------------------

INPUT_FILE <- "data.xlsx"	# input file, relative to the working directory

OUTPUT_DIR <- "output"	# results folder (created if missing)

\# Column names in the input file

COL_YEAR	<- "ANO_CMPT"	# year of procedure

COL_PROC	<- "PROC_REA...28" # procedure description / code

COL_REGION <- "Região_Hosp." # region (any stratifier; a "Brazil" total is added)

\# Keywords used to classify procedures (case-insensitive, partial match)

KW_MASTECTOMY	<- "Mastectomia"

KW_RECONSTRUCTION <- "Reconstru"

\# Interrupted time series parameters

INTERVENTION_YEAR <- 2013	# first year the intervention is in effect

COVID_YEARS	<- 2020:2022 # excluded in the pandemic sensitivity analysis

INTERVENTION_LAG <- 1	# +/- years tested in the lag sensitivity analysis

dir.create(OUTPUT_DIR, showWarnings = FALSE, recursive = TRUE)

\# ---------------------------------------------------------------------------

\# 2) Read and classify procedures

\# ---------------------------------------------------------------------------

raw <- readxl::read_excel(INPUT_FILE) %>%

mutate(

year	= as.numeric(.data[[COL_YEAR]]),

region	= as.character(.data[[COL_REGION]]),

procedure = as.character(.data[[COL_PROC]]),

group = case_when(

str_detect(procedure, regex(KW_MASTECTOMY, ignore_case = TRUE)) ~ "Mastectomy",

str_detect(procedure, regex(KW_RECONSTRUCTION, ignore_case = TRUE)) ~ "Reconstruction",

TRUE ~ NA_character_

)

) %>%

filter(!is.na(group), !is.na(year), !is.na(region))

\# ---------------------------------------------------------------------------

\# 3) Descriptive analysis (counts, rates, overall trend)

\# ---------------------------------------------------------------------------

\# Counts by year, region and group, plus a pooled "Brazil" stratum.

counts <- bind_rows(raw, mutate(raw, region = "Brazil")) %>%

count(year, region, group, name = "n")

\# Reconstruction as a share of all mastectomy-related procedures.

reconstruction_rate <- counts %>%

group_by(year, region) %>%

summarise(

total	= sum(n),

reconstruction	= sum(n[group == "Reconstruction"]),

reconstruction_rate = reconstruction / total,

.groups = "drop"

)

\# Reconstruction relative to mastectomy.

recon_vs_mastectomy <- counts %>%

filter(group %in% c("Mastectomy", "Reconstruction")) %>%

pivot_wider(names_from = group, values_from = n, values_fill = 0) %>%

mutate(

prop_reconstruction	= Reconstruction / (Reconstruction + Mastectomy),

ratio_recon_mastectomy = Reconstruction / Mastectomy

)

\# Overall direction of change (first vs last year) per region.

trend <- recon_vs_mastectomy %>%

group_by(region) %>%

arrange(year) %>%

summarise(

year_first = min(year),

year_last	= max(year),

ratio_first = first(ratio_recon_mastectomy),

ratio_last = last(ratio_recon_mastectomy),

direction = case_when(

ratio_last > ratio_first ~ "Increase",

ratio_last < ratio_first ~ "Decrease",

TRUE ~ "Stable"

),

.groups = "drop"

)

\# ---------------------------------------------------------------------------

\# 4) Annual series for the ITS (pooled "Brazil")

\#	Outcome = reconstruction count; offset = total mastectomy-related procedures.

\# ---------------------------------------------------------------------------

series <- counts %>%

filter(region == "Brazil", group %in% c("Mastectomy", "Reconstruction")) %>%

pivot_wider(names_from = group, values_from = n, values_fill = 0) %>%

rename(n_mastectomy = Mastectomy, n_reconstruction = Reconstruction) %>%

mutate(n_total = n_mastectomy + n_reconstruction) %>%

filter(n_total > 0) %>%

arrange(year)

\# ---------------------------------------------------------------------------

\# 5) ITS variable coding and helper functions

\#	time : 0 in the first year (reference year), +1 per calendar year

\#	level: 0 before the intervention, 1 from the intervention onward

\#	trend: 0 before; 1, 2, 3, ... from the intervention (slope change)

\# ---------------------------------------------------------------------------

make_its <- function(d, intervention_year) {

d %>%

arrange(year) %>%

mutate(

time	= year - min(year),

level	= as.integer(year >= intervention_year),

trend	= ifelse(year >= intervention_year, year - intervention_year + 1L, 0L),

offset_log = log(n_total)

)

}

series	<- make_its(series, INTERVENTION_YEAR)

REFERENCE_YEAR <- min(series$year)

\# Model equation:

\#	log E(reconstruction_t) = b0 + b1*time + b2*level + b3*trend + log(total_t)

its_formula <- n_reconstruction ~ time + level + trend + offset(offset_log)

\# Rate-ratio (IRR) table with Wald 95% CI from a variance matrix V.

rr_table <- function(m, V = NULL) {

b <- coef(m); if (is.null(V)) V <- vcov(m); se <- sqrt(diag(V))

data.frame(

term	= names(b),

IRR	= exp(b),

CI_low = exp(b - 1.96 * se),

CI_high= exp(b + 1.96 * se),

p	= 2 * pnorm(-abs(b / se)),

row.names = NULL

)

}

\# Newey-West / HAC variance for a Poisson GLM (Bartlett kernel, lag L), base R.

\# Equivalent to lmtest::coeftest(m, vcov. = sandwich::NeweyWest(m, lag = L,

\# prewhite = FALSE)). Reported as a robustness check only.

hac_poisson <- function(m, L = 1) {

X <- model.matrix(m); mu <- fitted(m); y <- m$y

u <- X * (y - mu); nn <- nrow(X)

bread <- solve(t(X) %*% (X * mu))	# (X' diag(mu) X)^-1

meat <- t(u) %*% u

if (L >= 1) for (l in 1:L) {

w <- 1 - l / (L + 1)

Gl <- t(u[(l + 1):nn, , drop = FALSE]) %*% u[1:(nn - l), , drop = FALSE]

meat <- meat + w * (Gl + t(Gl))

}

V <- bread %*% meat %*% bread

dimnames(V) <- list(colnames(X), colnames(X))

V

}

\# Fit the main (negative binomial) model on a given series + intervention year

\# and return the level/trend IRRs (used for the sensitivity table).

its_summary <- function(d, intervention_year, label) {

s <- make_its(d, intervention_year)

m <- tryCatch(

MASS::glm.nb(its_formula, data = s),

error = function(e) glm(its_formula, family = poisson, data = s)

)

tab <- rr_table(m)

pick <- function(t) tab[tab$term == t, c("IRR", "CI_low", "CI_high", "p")]

lv <- pick("level"); tr <- pick("trend")

data.frame(

analysis = label, n_years = nrow(s),

level_IRR = lv$IRR, level_CI_low = lv$CI_low, level_CI_high = lv$CI_high, level_p = lv$p,

trend_IRR = tr$IRR, trend_CI_low = tr$CI_low, trend_CI_high = tr$CI_high, trend_p = tr$p,

row.names = NULL

)

}

\# ---------------------------------------------------------------------------

\# 6) Main model and diagnostics

\# ---------------------------------------------------------------------------

m_poisson <- glm(its_formula, family = poisson, data = series) # for overdispersion check

m_nb	<- MASS::glm.nb(its_formula, data = series)	# MAIN model

m_qpois	<- glm(its_formula, family = quasipoisson, data = series)	# robustness

main_coefficients <- rr_table(m_nb)

cat("\n== Main model (negative binomial): IRR (95% CI) ==\n")

print(main_coefficients, digits = 4)

\## 6.1 Overdispersion

dispersion_poisson <- sum(residuals(m_poisson, "pearson")^2) / df.residual(m_poisson)

phi_quasipoisson	<- summary(m_qpois)$dispersion

cat(sprintf("\nOverdispersion: Pearson dispersion (Poisson) = %.2f | quasi-Poisson phi = %.2f\n",

dispersion_poisson, phi_quasipoisson))

cat(sprintf("AIC: Poisson = %.1f | negative binomial = %.1f | theta = %.2f\n",

AIC(m_poisson), AIC(m_nb), m_nb$theta))

\## 6.2 Residual autocorrelation (ACF / PACF + Durbin-Watson) on the main model

res_dev <- residuals(m_nb, type = "deviance")

dw	<- sum(diff(res_dev)^2) / sum(res_dev^2)	# ~2 indicates no autocorrelation

acf_r	<- acf(res_dev, plot = FALSE)

pacf_r <- pacf(res_dev, plot = FALSE)

cat(sprintf("\nDurbin-Watson = %.3f | ACF lag1 = %.3f | ACF lag2 = %.3f | PACF lag1 = %.3f\n",

dw, acf_r$acf[2], acf_r$acf[3], pacf_r$acf[1]))

png(file.path(OUTPUT_DIR, "fig_acf_pacf.png"), width = 1100, height = 500, res = 130)

op <- par(mfrow = c(1, 2))

acf(res_dev, main = "ACF of residuals")

pacf(res_dev, main = "PACF of residuals")

par(op); dev.off()

\## 6.3 Newey-West (HAC) standard errors -- robustness check (computed on Poisson fit)

newey_west <- rr_table(m_poisson, hac_poisson(m_poisson, L = 1))

cat("\n== Newey-West (HAC, lag 1) robustness check: IRR (95% CI) ==\n")

print(newey_west, digits = 4)

\## 6.4 Influential observations (Cook's distance on the main model)

cook <- cooks.distance(m_nb)

thr	<- 4 / nrow(series)

influential_years <- series$year[cook > thr]

cat(sprintf("\nInfluential years (Cook's D > %.3f): %s\n",

thr, ifelse(length(influential_years) == 0, "none",

paste(influential_years, collapse = ", "))))

\## 6.5 Fit statistics and diagnostics table

pseudo_r2 <- 1 - m_nb$deviance / m_nb$null.deviance

diagnostics <- tibble::tibble(

metric = c("Pearson dispersion (Poisson)", "Quasi-Poisson phi",

"AIC Poisson", "AIC negative binomial", "Negative binomial theta",

"Residual deviance (NB)", "Residual df (NB)", "Pseudo-R2 (deviance, NB)",

"Durbin-Watson", "ACF lag 1", "ACF lag 2", "PACF lag 1",

"Influential years (Cook's D > 4/n)"),

value = c(round(dispersion_poisson, 2), round(phi_quasipoisson, 2),

round(AIC(m_poisson), 1), round(AIC(m_nb), 1), round(m_nb$theta, 2),

round(m_nb$deviance, 2), df.residual(m_nb), round(pseudo_r2, 3),

round(dw, 3), round(acf_r$acf[2], 3), round(acf_r$acf[3], 3),

round(pacf_r$acf[1], 3), length(influential_years))

)

\## 6.6 Alternative model specifications (level and trend, side by side)

pick <- function(tab, term) tab[tab$term == term, c("IRR", "CI_low", "CI_high", "p")]

alt_models <- bind_rows(

cbind(model = "Negative binomial", parameter = "Level change", pick(rr_table(m_nb), "level")),

cbind(model = "Negative binomial", parameter = "Trend change", pick(rr_table(m_nb), "trend")),

cbind(model = "Poisson", parameter = "Level change", pick(rr_table(m_poisson), "level")),

cbind(model = "Poisson", parameter = "Trend change", pick(rr_table(m_poisson), "trend")),

cbind(model = "Quasi-Poisson", parameter = "Level change", pick(rr_table(m_qpois), "level")),

cbind(model = "Quasi-Poisson", parameter = "Trend change", pick(rr_table(m_qpois), "trend")),

cbind(model = "Newey-West (HAC)", parameter = "Level change", pick(newey_west, "level")),

cbind(model = "Newey-West (HAC)", parameter = "Trend change", pick(newey_west, "trend"))

)

\## 6.7 ITS figure: observed vs fitted vs counterfactual (no intervention)

counterfactual <- series %>% mutate(level = 0L, trend = 0L)

series <- series %>%

mutate(

rate_observed	= n_reconstruction / n_total,

rate_fitted	= predict(m_nb, type = "response") / n_total,

rate_counterfactual = predict(m_nb, newdata = counterfactual, type = "response") / n_total

)

png(file.path(OUTPUT_DIR, "fig_its_fit.png"), width = 1100, height = 650, res = 130)

plot(series$year, series$rate_observed, pch = 19, xlab = "Year",

ylab = "Reconstruction rate", main = "Interrupted time series: observed, fitted, counterfactual",

ylim = range(c(series$rate_observed, series$rate_fitted, series$rate_counterfactual)))

lines(series$year, series$rate_fitted, lwd = 2)

lines(series$year, series$rate_counterfactual, lwd = 2, lty = 2)

abline(v = INTERVENTION_YEAR - 0.5, col = "grey50", lty = 3)

legend("topleft", bty = "n",

legend = c("Observed", "Fitted (negative binomial)", "Counterfactual (no intervention)"),

pch = c(19, NA, NA), lty = c(NA, 1, 2), lwd = c(NA, 2, 2))

dev.off()

\# ---------------------------------------------------------------------------

\# 7) Sensitivity analyses (negative binomial)

\#	- excluding the pandemic years

\#	- shifting the intervention point by +/- INTERVENTION_LAG year(s)

\# ---------------------------------------------------------------------------

sensitivity <- bind_rows(

its_summary(series, INTERVENTION_YEAR, "Main analysis (full series)"),

its_summary(filter(series, !(year %in% COVID_YEARS)), INTERVENTION_YEAR,

sprintf("Excluding pandemic years (%d-%d)", min(COVID_YEARS), max(COVID_YEARS))),

its_summary(series, INTERVENTION_YEAR - INTERVENTION_LAG,

sprintf("Intervention lag, -%d year", INTERVENTION_LAG)),

its_summary(series, INTERVENTION_YEAR + INTERVENTION_LAG,

sprintf("Intervention lag, +%d year", INTERVENTION_LAG))

)

cat("\n== Sensitivity analyses (negative binomial): level and trend IRR ==\n")

print(sensitivity, digits = 3)

\# ---------------------------------------------------------------------------

\# 8) Export results

\# ---------------------------------------------------------------------------

writexl::write_xlsx(

list(

"descr_counts"	= counts,

"descr_reconstruction_rate" = reconstruction_rate,

"descr_recon_vs_mastectomy" = recon_vs_mastectomy,

"descr_trend"	= trend,

"its_series"	= series,

"its_main_NB"	= main_coefficients,

"its_diagnostics"	= diagnostics,

"its_alt_models"	= alt_models,

"its_newey_west"	= newey_west,

"its_sensitivity"	= sensitivity

),

file.path(OUTPUT_DIR, "reconstruction_results.xlsx")

)

\# ---------------------------------------------------------------------------

\# 9) Reproducibility: record R and package versions

\# ---------------------------------------------------------------------------

writeLines(capture.output(sessionInfo()), file.path(OUTPUT_DIR, "sessionInfo.txt"))

cat("\n--- DONE ---\n")

cat("Reference year:", REFERENCE_YEAR, "| Intervention year:", INTERVENTION_YEAR,

"| Years in series:", nrow(series), "\n")

cat("Outputs written to: ", normalizePath(OUTPUT_DIR), "\n", sep = "")

print(sessionInfo())
