# Supplementary material for: Breast reconstruction and breast cancer surgery in Brazil’s public health system: a nationwide interrupted time-series analysis, 2008–2024
Source: BMC Womens Health. 2026 Aug 5;26:574. doi: 10.1186/s12905-026-04666-x (PMC13726083; doi:10.1186/s12905-026-04666-x)
Supplement: Supplementary file 2 [file 12905_2026_4666_MOESM2_ESM.docx]

Supplementary Table S1

Annual volume of breast reconstruction hospital admissions according to SIGTAP procedure code, Brazil, 2008–2024.

| **Year** | **0410010090 Non-aesthetic female breast reconstruction surgery** | **0410010090 Post-mastectomy reconstructive breast surgery with implant** | **0410010219 Post-total mastectomy breast reconstruction** | **Total (all procedures)** |
| --- | --- | --- | --- | --- |
| **2008** | 29 | 1.354 | - | 1.383 |
| **2009** | - | 1.301 | - | 1.301 |
| **2010** | - | 1.358 | - | 1.358 |
| **2011** | - | 1.344 | - | 1.344 |
| **2012** | - | 1.394 | - | 1.394 |
| **2013** | - | 1.537 | - | 1.537 |
| **2014** | - | 1.711 | - | 1.711 |
| **2015** | 203 | 1.507 | - | 1.710 |
| **2016** | 421 | 1.371 | - | 1.792 |
| **2017** | 553 | 1.337 | - | 1.890 |
| **2018** | 576 | 1.304 | - | 1.880 |
| **2019** | 641 | 1.453 | - | 2.094 |
| **2020** | 373 | 821 | - | 1.194 |
| **2021** | 464 | 800 | - | 1.264 |
| **2022** | 728 | 1.155 | - | 1.883 |
| **2023** | 836 | 1.341 | 539 | 2.716 |
| **2024** | 777 | 1.204 | 1.616 | 3.597 |
| **Total** | 5.601 | 22.292 | 2.155 | 30.048 |

Note: "—" indicates zero hospitalizations.

Supplementary Table S2. Annual approved reimbursement values for breast cancer-related surgical procedures and breast reconstruction hospital admissions (Brazil, 2008–2024).

| **Section A: Mean reimbursement per hospital admission (nominal values)** |  |  |  |  |  |  |  |  |  |  |  |  |  |  |  |  |  |  |
| --- | --- | --- | --- | --- | --- | --- | --- | --- | --- | --- | --- | --- | --- | --- | --- | --- | --- | --- |
| **Year** | 2008 | 2009 | 2010 | 2011 | 2012 | 2013 | 2014 | 2015 | 2016 | 2017 | 2018 | 2019 | 2020 | 2021 | 2022 | 2023 | 2024 | Overall mean (2008–2024) |
| **Breast cancer-related surgical procedures (R$)** | 639.31 | 689.07 | 712.11 | 720.18 | 732.89 | 1,948.41 | 2,113.19 | 2,228.62 | 2,183.22 | 2,233.06 | 2,257.63 | 2,207.51 | 2,284.59 | 2,347.55 | 2,314.09 | 2,321.24 | 2,564.73 | 1,750.02 |
| **Breast reconstruction (R$)** | 832.47 | 836.03 | 854.82 | 873.88 | 831.38 | 855.34 | 878.67 | 916.81 | 924.94 | 935.45 | 951.25 | 974.73 | 994.81 | 1,069.55 | 1,020.83 | 2,132.83 | 3,403.51 | 1,326.92 |
| **Annual IPCA (%) *** | 5.91 | 4.31 | 5.91 | 6.50 | 5.84 | 5.91 | 6.41 | 10.67 | 6.29 | 2.95 | 3.75 | 4.31 | 4.52 | 10.06 | 5.79 | 4.62 | 4.83 | — |
|  |  |  |  |  |  |  |  |  |  |  |  |  |  |  |  |  |  |  |
| **Section B: Mean reimbursement per hospital admission – constant 2024 Brazilian reais (R$)** |  |  |  |  |  |  |  |  |  |  |  |  |  |  |  |  |  |  |
| **Year** | 2008 | 2009 | 2010 | 2011 | 2012 | 2013 | 2014 | 2015 | 2016 | 2017 | 2018 | 2019 | 2020 | 2021 | 2022 | 2023 | 2024 | Overall mean (2008–2024) |
| **Breast cancer-related surgical procedures (R$)** | 1,569.39 | 1,621.64 | 1,582.35 | 1,502.61 | 1,444.76 | 3,626.60 | 3,696.37 | 3,522.43 | 3,246.47 | 3,225.43 | 3,143.06 | 2,946.30 | 2,917.31 | 2,723.70 | 2,533.70 | 2,433.94 | 2,564.73 | 2,606.14 |
| **Breast reconstruction (R$)** | 2,043.56 | 1,967.50 | 1,899.46 | 1,823.30 | 1,638.91 | 1,592.05 | 1,536.96 | 1,449.06 | 1,375.40 | 1,351.17 | 1,324.32 | 1,300.94 | 1,270.32 | 1,240.93 | 1,119.58 | 2,235.85 | 3,403.51 | 1,680.75 |
|  |  |  |  |  |  |  |  |  |  |  |  |  |  |  |  |  |  |  |
| **Section C: Mean reimbursement per hospital admission – constant 2024 US dollars (US$) **** |  |  |  |  |  |  |  |  |  |  |  |  |  |  |  |  |  |  |
| **Year** | 2008 | 2009 | 2010 | 2011 | 2012 | 2013 | 2014 | 2015 | 2016 | 2017 | 2018 | 2019 | 2020 | 2021 | 2022 | 2023 | 2024 | Overall mean (2008–2024) |
| **Breast cancer-related surgical procedures (US$)** | 291.17 | 300.86 | 293.57 | 278.78 | 268.04 | 672.84 | 685.78 | 653.51 | 602.31 | 598.41 | 583.13 | 546.62 | 541.25 | 505.33 | 470.86 | 451.46 | 475.83 | 483.51 |
| **Breast reconstruction (US$)** | 379.14 | 365.03 | 352.40 | 338.27 | 304.07 | 295.37 | 285.15 | 268.84 | 255.18 | 250.68 | 245.70 | 241.36 | 235.68 | 230.23 | 207.71 | 414.81 | 631.45 | 311.83 |
|  |  |  |  |  |  |  |  |  |  |  |  |  |  |  |  |  |  |  |
| **Section D: Number of hospital admissions (n)** |  |  |  |  |  |  |  |  |  |  |  |  |  |  |  |  |  |  |
| **Year** | 2008 | 2009 | 2010 | 2011 | 2012 | 2013 | 2014 | 2015 | 2016 | 2017 | 2018 | 2019 | 2020 | 2021 | 2022 | 2023 | 2024 | Total (2008–2024) |
| **Breast cancer-related surgical procedures** | 20,726 | 20,899 | 19,735 | 18,883 | 19,841 | 19,224 | 18,331 | 18,876 | 19,142 | 18,846 | 18,495 | 17,805 | 15,100 | 14,648 | 16,023 | 17,095 | 18,706 | 312,375 |
| **Breast reconstruction** | 1,383 | 1,301 | 1,358 | 1,344 | 1,394 | 1,537 | 1,711 | 1,710 | 1,792 | 1,890 | 1,880 | 2,094 | 1,194 | 1,264 | 1,883 | 2,716 | 3,597 | 30,048 |

Supplementary Table S3. Distribution of breast cancer-related surgical admissions and breast reconstruction admissions according to ICD-10 diagnosis (C50 and D05), Brazil, 2008–2024.

| Procedure, ICD-10 | Breast cancer-related surgical procedures, C50 | Breast cancer-related surgical procedures, D05 | Breast cancer-related surgical procedures, Total | Reconstruction, C50 | Reconstruction, D05 | Reconstruction, Total | Breast cancer-related surgical procedures, C50 (%) | Reconstruction, C50 (%) | Breast cancer-related surgical procedures, D05 (%) | Reconstruction, D05 (%) |
| --- | --- | --- | --- | --- | --- | --- | --- | --- | --- | --- |
| 2008 | 19,520 | 1,206 | 20,726 | 1,381 | 2 | 1,383 | 94.2% | 99.9% | 5.8% | 0.1% |
| 2009 | 19,551 | 1,348 | 20,899 | 1,301 | 0 | 1,301 | 93.5% | 100.0% | 6.5% | 0.0% |
| 2010 | 18,534 | 1,201 | 19,735 | 1,358 | 0 | 1,358 | 93.9% | 100.0% | 6.1% | 0.0% |
| 2011 | 17,699 | 1,184 | 18,883 | 1,344 | 0 | 1,344 | 93.7% | 100.0% | 6.3% | 0.0% |
| 2012 | 18,775 | 1,066 | 19,841 | 1,394 | 0 | 1,394 | 94.6% | 100.0% | 5.4% | 0.0% |
| 2013 | 18,191 | 1,033 | 19,224 | 1,537 | 0 | 1,537 | 94.6% | 100.0% | 5.4% | 0.0% |
| 2014 | 17,119 | 1,212 | 18,331 | 1,711 | 0 | 1,711 | 93.4% | 100.0% | 6.6% | 0.0% |
| 2015 | 17,232 | 1,644 | 18,876 | 1,709 | 1 | 1,710 | 91.3% | 99.9% | 8.7% | 0.1% |
| 2016 | 17,399 | 1,743 | 19,142 | 1,791 | 1 | 1,792 | 90.9% | 99.9% | 9.1% | 0.1% |
| 2017 | 17,275 | 1,571 | 18,846 | 1,887 | 3 | 1,890 | 91.7% | 99.8% | 8.3% | 0.2% |
| 2018 | 16,994 | 1,501 | 18,495 | 1,867 | 13 | 1,880 | 91.9% | 99.3% | 8.1% | 0.7% |
| 2019 | 16,302 | 1,503 | 17,805 | 2,082 | 12 | 2,094 | 91.6% | 99.4% | 8.4% | 0.6% |
| 2020 | 13,987 | 1,113 | 15,100 | 1,191 | 3 | 1,194 | 92.6% | 99.7% | 7.4% | 0.3% |
| 2021 | 13,598 | 1,050 | 14,648 | 1,262 | 2 | 1,264 | 92.8% | 99.8% | 7.2% | 0.2% |
| 2022 | 14,703 | 1,320 | 16,023 | 1,875 | 8 | 1,883 | 91.8% | 99.6% | 8.2% | 0.4% |
| 2023 | 15,796 | 1,299 | 17,095 | 2,697 | 19 | 2,716 | 92.4% | 99.3% | 7.6% | 0.7% |
| 2024 | 17,138 | 1,568 | 18,706 | 3,560 | 37 | 3,597 | 91.6% | 99.0% | 8.4% | 1.0% |
| Total | 289,813 | 22,562 | 312,375 | 29,947 | 101 | 30,048 | 92.8% | 99.7% | 7.2% | 0.3% |

Supplementary Table S4. Interrupted time-series of breast reconstruction: sensitivity analysis restricted to invasive carcinoma (ICD-10 C50)

| **Analysis** | **Procedures, n** | **Level change, IRR (95% CI)** | **p** | **Trend change, IRR (95% CI)** | **p** | **C50 share** |
| --- | --- | --- | --- | --- | --- | --- |
| **Full cohort (C50 + D05)** | 342,423 | 1.01 (0.96–1.07) | 0.59 | 1.04 (1.02–1.05) | <0.001 | — |
| **C50 only** | 319,760 | 1.03 (0.98–1.09) | 0.22 | 1.04 (1.02–1.05) | <0.001 | Mast. 92.8% Rec. 99.7% |

IRR, incidence rate ratio; CI, confidence interval; ICD-10, International Classification of Diseases, 10th revision. Reconstruction counts were modelled with a log link and an offset for the annual total of mastectomy-related procedures; the level change is the immediate post-intervention shift and the trend change is the difference in the post-intervention slope. C50 accounted for 92.8% of mastectomies and 99.7% of reconstructions (D05: 7.2% and 0.3%, respectively). Restricting the cohort to C50 left both estimates essentially unchanged.
